# Anti-Inflammatory and Chemopreventive Effects of *Bryophyllum pinnatum* (Lamarck) Leaf Extract in Experimental Colitis Models in Rodents

**DOI:** 10.3389/fphar.2020.00998

**Published:** 2020-07-29

**Authors:** Anderson Wilbur Lopes Andrade, Gerlane Coelho Bernardo Guerra, Daline Fernandes de Souza Araújo, Raimundo Fernandes de Araújo Júnior, Aurigena Antunes de Araújo, Thaís Gomes de Carvalho, Júlia Morais Fernandes, Patrícia Diez-Echave, Laura Hidalgo-García, Maria Elena Rodriguez-Cabezas, Julio Gálvez, Silvana Maria Zucolotto

**Affiliations:** ^1^Department of Biophysics and Pharmacology, Federal University of Rio Grande do Norte, Natal, Brazil; ^2^Health Science Center, Postgraduate Program in Drug Development and Technological Innovation, Federal University of Rio Grande do Norte, Natal, Brazil; ^3^Health Sciences College of Trairi, Federal University of Rio Grande do Norte, Santa Cruz, Brazil; ^4^Postgraduate Program in Health Science, Federal University of Rio Grande do Norte (UFRN), Natal, Brazil; ^5^Postgraduate Program in Functional and Structural Biology, Department of Morphology, Federal University of Rio Grande do Norte (UFRN), Natal, Brazil; ^6^Instituto de Investigación Biosanitaria de Granada (ibs.GRANADA), University of Granada, Granada, Spain; ^7^CIBER-EHD, Department of Pharmacology, Center for Biomedical Research (CIBM), University of Granada, Granada, Spain

**Keywords:** 2.4-dinitrobenzene sulfonic acid, dextran sulfate sodium, herbal drug, flavonoid, inflammatory bowel diseases, immune response, oxidative stress

## Abstract

Inflammatory bowel diseases, mainly ulcerative colitis and Crohn’s disease are characterized by chronic inflammation in the intestine. Currently several therapeutic strategies available to treat inflammatory bowel diseases. Though, most treatments can be associated with serious adverse effects what justifies the search for new treatments. In this sense, we highlight the interest in herbal products rich in bioactive compounds which immunomodulatory and antioxidant properties as is the case of *Bryophyllum pinnatum* (Crassulaceae). This plant is used in traditional medicine in Brazil for treating inflammatory diseases. We hypothesized that hydroethanolic *B. pinnatum* leaf extract has intestinal anti-inflammatory effects on two experimental colitis models: 2.4-dinitrobenzene sulfonic acid (DNBS) in rats, and dextran sulfate sodium (DSS) in mice. Ultra-fast liquid chromatography method used for the quantification of the main compounds indicated good linearity, specificity, selectivity, precision, robustness and accuracy. The major flavonoids (mg/g of the extract) quantified were: quercetin 3-*O*-α-L-arabinopyranosyl-(1→2)-α-L-rhamnopyranoside (35.56 ± 0.086 mg/g), kaempferol 3-*O*-α-L-arabinopyranosyl-(1→2)-α-L-rhamnopyranoside (4.66 ± 0.076 mg/g) and quercetin-3-*O*-rhamnopyranoside (4.56 ± 0.026 mg/g). The results obtained in the DNBS and DSS models indicate that extract has both chemopreventive and anti-inflammatory effects, observing a significant reduction in the disease activity index score, and less macroscopic and microscopic damage. The extract promoted downregulation of Toll-like receptor and *kappa* B p65 nuclear factor gene expression, leading to a reduction in pro-inflammatory and oxidative mediators, chemokines, and cell adhesion molecules. This immunomodulatory property was proposed that one of the possible action mechanisms of extract. An improvement in intestinal damage was also associated with a reduction in oxidative stress and infiltration of leukocytes, as evidenced by the reduction in malonaldialdehyde and myeloperoxidase activity and increase in total glutathione in the colonic tissue. Moreover, the extract improved the cytoarchitecture of the colonic tissue and the integrity of the intestinal epithelial barrier by restoring the expression of the proteins associated with mucosa protection. In view of the beneficial effects showed by the *B. pinnatum* leaf extract in preclinical rodent models of colitis there is the potential to conduct some future clinical studies to ensure safe and effective development of a phytotherapeutic treatment for human inflammatory bowel diseases.

## Introduction

Ulcerative colitis (UC) and Crohn’s disease (DC) are inflammatory bowel diseases (IBD) referring to chronic idiopathic diseases of the gastrointestinal tract, being characterized by chronic and recurrent inflammation of the intestine (Wu et al., 2020). Its etiology is not yet fully understood, but it is most likely related to genetic and environmental factors, changes in the composition of the intestinal microbiota and an exacerbated and abnormal immune response (Pallio et al., 2016). This altered immune response promotes an increase in inflammatory mediators responsible for generating and maintaining inflammation in IBD, including pro-inflammatory cytokines such as interleukin (IL)-1β, IL-6, IL-17, tumor necrosis factor (TNF) α, prostaglandins, and nitric oxide (NO). Previous studies have found strong evidence that increased expression of *kappa* B p65 nuclear factor (NF-κB p65), a transcription factor involved in controlling the expression of several genes linked to the inflammatory response, is followed by increased capacity of defense cells (neutrophils and macrophages) to produce and secrete TNF-α, IL-1β, and IL-6, chemokines [monocyte chemoattractant protein-1 (MCP-1) and macrophage inflammatory protein-2 (MIP-2)], cell adhesion molecules [type I intercellular adhesion molecules (ICAM-1)], eicosanoids, and increases the oxidative stress by reactive oxygen (ROS) and nitrogen (RNS) species on cells (Lu et al., 2018; Wang et al., 2018).

In IBD, chronic inflammation occurs because acute inflammatory mechanisms cannot eliminate tissue injury. In this process, germline-encoded pattern-recognition receptors (PRRs) are activated in both immune and non-immune cells. Classes of PRR families include Toll-like receptors (TLRs). TLRs are a family of highly conserved, mammalian PRRs which participate in activating the inflammatory response. TLRs can mediate the differentiation of T-cells into effector T helper (Th or CD4+) cells, including Th1, Th2, and Th17 cell types and proliferate macrophages, thus impairing the intestinal mucosa. Furthermore, signaling through TLRs activates an intracellular signaling cascade which leads to nuclear translocation of transcription factors such as NF-κB p65 (Chen et al., 2018). Nitric oxide synthase (NOS) in the inducible isoform (iNOS) is highly expressed by immune cells (e.g. macrophages) after activation of NF-κB p65 in response to many stimuli, including TNF-α, interferon gamma, IL-6, IL-1β, bacterial, and viral components. L-arginine then generates NO by oxidizing the amino acid, which exerts protective effects during acute experimental colitis. The high levels of iNOS and consequently NO released in the mucosa in IBD cause severe tissue damage and intensified inflammation (Soufli et al., 2016).

The intestinal epithelium forms the physical, protective, and host defense barrier against the harmful luminal microenvironment. The epithelium is covered by a single-cell layer composed of different subtypes of specialized intestinal epithelial cells (IEC) including goblet cells, Paneth cells enteroendocrine cells, enterocytes, M cells, cup cells, and Tuft cells (Garcia-Carbonell et al., 2019). These IEC, especially the goblet cells produce secretory [mucin type II (MUC-2)] and membrane-associated mucins [mucin type III (MUC-3)], villin (actin regulatory protein), and trefoil factors [trefoil factor 3 (TFF-3)], which are responsible for cell morphology and epithelial restitution (Algieri et al., 2013; Dorofeyev et al., 2013). Moreover, the junctional complexes cells in the mucosal epithelium forms physiologically active barrier mediated by a cluster of proteins, including zonula occludens type 1 (ZO-1) and occludin (tight junctions complex) that can change intestinal permeability (Poritz et al., 2007). Bioactive or epigenetic factors such as microbes, toxins, and pro-inflammatory cytokines, promoting a rupture in the barrier and loss of proper mucins (MUC-2 and MUC-3) secretion which leads to chronic inflammation in IBD (Okamoto and Watanabe, 2016; Garcia-Carbonell et al., 2019).

Patients with IBD present abdominal pain and bloody diarrhea, and the disease has a long course. Recurrence is common, along with severe complications and poor prognosis by pharmacological treatment (Wu et al., 2020). The pharmacological treatments for human IBD mainly comprise anti-inflammatory and immunosuppressive drugs, which can be linked to significant side effects, thus limiting their use (Pagnini et al., 2019). There is consequently a clear demand for new treatments which combine efficacy and safety, such as those of natural origin. Applications of medicinal plants in both the prevention and treatment of chronic inflammatory diseases has been widely described in the literature (Kim et al., 2018). Specifically regarding *Bryophyllum pinnatum*, its use has been recognized in *in vivo* studies to treat some inflammatory diseases and for healing wounds (dos Santos Nascimento et al., 2018; Fernandes et al., 2019).

*B. pinnatum* (Lamark) (synonym *B. calycinum* and *Kalanchoe pinnata* (Lamk) Pers.), Crassulaceae family, popularly known as “*saião*” and “*coirama*”, is widely used in traditional medicine worldwide, mainly in treating inflammatory, gastritis, and ulcer problems (The Plant List, 2010; Fernandes et al., 2019). *B. pinnatum* is rich in phenolic compounds, especially quercetin- and kaempferol-derived flavonoid glycosides, which are responsible for some of these aforementioned biological activities, and other secondary metabolites such as terpenes, steroids, and bufadienolides (Muzitano et al., 2006; Cruz et al., 2012; Fernandes et al., 2016; de Araújo et al., 2019). In acute toxicity studies (14 days; using albino Swiss and BALB/c mice) with ethanolic and aqueous *B. pinnatum* extracts (administered orally) conducted as per Organization for Economic Co-operation and Development (OECD) guidelines, no mortality, noticeable behavioral, or biochemistry analysis changes were observed in any of the tested groups. The extracts were found to be safe up to 2000 mg/kg body weight with no mortality, thus the 1/8 and 1/4 doses (i.e., 250 mg/kg and 500 mg/kg) can be selected for all further *in vivo* studies (Torres-Santos et al., 2003; Afzal et al., 2013).

It is important to mention that this species is included on the National List of Medicinal Plants of Interest to the Brazilian Public Health System (*RENISUS*), which is a report published by the Brazilian health ministry which includes medicinal plants which can be used in the near future by the population and for developing phytotherapeutic medicines (Brasil, 2009). Although *B. pinnatum* is widely used by traditional medicine practitioners, its chemical constituents have not been fully elucidated. Few investigations have involved isolating and elucidating its bioactive compounds until now, but most of the studies have indicated the presence of glycosylated flavonoids, with acetylated rhamnose sugar linked to different positions of aglycone (Fernandes et al., 2019).

Among the constituents analyzed in *B. pinnatum* leaf, quercetin 3-*O*-α-L-arabinopyranosyl-(1→2)-α-L-rhamnopyranoside (**Bp1**) was the first compound isolated for this specie (Muzitano et al., 2006), and it is the major compound reported in literature (Sobreira et al., 2017; dos Santos Nascimento et al., 2018). Fernandes et al. (2019) and Ferreira et al. (2014) reported other compounds already described for *B. pinnatum*: kaempferol 3-*O*-α-L-arabinopyranosyl-(1→2)-α-L-rhamnopyranoside (**Bp2**) and quercetin-3-*O*-rhamnopyranoside (**Bp3**). These three compounds are considered as potential candidates for specific markers of this species and can be used in authenticating its raw material (dos Santos Nascimento et al., 2018). In addition, they present relevant preclinical pharmacological evidence, especially antioxidant, anti-inflammatory, and immunosuppressive activities (Muzitano et al., 2006; Ferreira et al., 2014; Sobreira et al., 2017).

It is believed that plants that are rich in phenolic compounds (e.g., quercetin and kaempferol) like *B. pinnatum* could constitute possible alternatives in preventing and treating chronic inflammation, as well as autoimmune abnormalities (Samsami-kor et al., 2015). Clinical studies have demonstrated that phenolic supplementation can improve quality of life in patients with active mild to moderate UC, at least partially through a reduction of inflammatory markers (Hanai et al., 2006; Lang et al., 2015; Samsamikor et al., 2016). Thus, the objective of this study was to evaluate the intestinal anti-inflammatory effects of hydroethanolic extract from *B. pinnatum* leaf (HEBP) in two experimental models of intestinal inflammation: 2.4-dinitrobenzene sulfonic (DNBS) model in rats, and oral dextran sulfate sodium (DSS) in mice.

## Material and Methods

### Reagents and Plant Material

Most of the chemicals used were purchased from Sigma-Aldrich chemical (São Paulo, Brazil; Madrid, Spain) unless otherwise stated. **Bp1** (99.3% purity), **Bp2** (98.9% purity) and **Bp3** (99.6% purity) were previously isolated from *Bryophyllum pinnatum* in our laboratory and identified by nuclear magnetic resonance (NMR) and mass spectrometry (MS). The purity of the isolated compounds was determined by software of ultra-fast liquid chromatography coupled with a diode-array detector (UFLC-DAD) (Shimadzu Model LC-20AD, with DAD detector model SPD-M20A). NMR analysis of each sample also showed integration signals related to one compound (*results not shown*). *Bryophyllum pinnatum* (available at www.theplantlist.org) leaf were collected from the “*Escola Agrícola de Jundiaí*” in Macaíba city, Rio Grande do Norte state, Brazil, in March of 2017. The botanical identification voucher specimen (No. 57335) was deposited at the herbarium of the Bioscience Center of the Federal University of Rio Grande do Norte. The collection was authorized by the Brazilian Authorization and Biodiversity Information System (SISBIO process No. 35017) and the research was authorized by the National System for the Management of Genetic Heritage and Associated Traditional Knowledge (SISGEN process No. A7EA798).

### HEBP Preparation and Quantitative Analyses of Major Flavonoids by UFLC-DAD

Fresh *B. pinnatum* leaf (3.8 kg) were processed by turbo extraction with ethanol:water (1:1, v/v) for 5 min to obtain the HEBP. The extract was filtered and concentrated on a rotoevaporator (model V-700, Buchi) and freeze-dried (extraction yield of 1.87%). One part of each freeze-dried HEBP were analyzed by UFLC-DAD (Shimadzu Model LC-20AD, with DAD detector model SPD-M20A). The method used was validated according to the parameters of Brazilian legislation No. 166 from 24 July 2017, which is aligned with the Guideline Q2 (R1) do International Conference on Harmonisation of Technical Requirements for Registration of Pharmaceuticals for Human Use (ICH, 2005): selectivity, linearity, limits of detection (LOD) and quantification (LOQ), precision, accuracy, content, and robustness. Prior to starting the validation, the system suitability of the developed method was verified according to the parameters established in the United States Pharmacopeia (USP) to guarantee resolution (R > 1.5), column efficiency (P > 2,000), tailing factor (T ≤ 2.0), and capacity factor (k’ ≥ 2.0) for each peak to be subsequently quantified (USP, 2003).

All analyses were performed in triplicate and the relative standard deviation was calculated. The samples were resuspended in 1:1 methanol:water (v/v), and the final concentration was 2 mg/mL for the extracts. A Phenomenex Kinetex Core-Shell RP-18 column (150x4.6 mm, 2.6 μm particle size) equipped with a Phenomenex security guard column (4.0x2.0 mm ID) was used. The eluents were: (A) trifluoroacetic acid 0.3% and (B) acetonitrile. The following gradient (v/v) was applied: 7–15% B, 0–3 min; 15–20% B, 3–12 min; 20–22% B, 12–30 min; with 30 min total analysis time. Flow elution was 0.7 ml/min, and 12 μl of each sample was injected. The UV-DAD detector was programmed for wavelength 200–500 nm and the chromatogram was plotted at 254 nm and 340 nm. *B. pinnatum* is a plant rich in flavonoids, then the chromatogram was analyzed at UV 340 nm, but also recorded at UV 254 nm to verify the presence or absence of other possible secondary metabolites (Aparicio and Harwood, 2013).

### *In Vivo* Studies

A total of 72 animals were included in this investigation. Female Wistar rats (180 ± 20 g, 6-8 weeks old) were used for the colitis induced by intra-colonic administration of DNBS (25 mg) in a 50% (v/v) ethanol/water solution (Morampudi et al., 2014). The animals were fasted overnight and anesthetized with ketamine (50 mg/kg) and xylazine (5 mg/kg) by intraperitoneal (IP) route. Male C57BL/6J mice (7–9 weeks old) were used for colitis induced by oral administration of 3% (w/v) DSS (36–50 KDa, MP Biomedicals, Ontario, USA) (Vezza et al., 2017). All animals were raised in accordance with the National Institute of Health Guide for Laboratory Animals. Rodents were acclimated for 7 days prior to experimentation and housed under standard environment conditions at 20–25°C and 12 h dark/light cycle and had free access to potable water (*ad libitum)* and standard food. These procedures were approved by the Ethics Committee of Laboratory Animals of the University of Granada (Spain) (Ref. No. CEEA-2010-286) and by the Federal University of Rio Grande do Norte (CEUA No. 26/2016 and No. 60/2017).

#### DNBS-Induced Colitis: Experimental Design and Treatment Protocol

Female Wistar rats (n = 40) were randomized into five groups (n = 8/groups): control groups (non-colitic and colitic: DNBS), HEBP (250 mg/kg and 500 mg/kg) and sulfasalazine (SSZ; 250 mg/kg). The vehicle (water; control groups), the extract, and the sulfasalazine were orally administered for two days before the colitis induction and three days after the induction. On day 3, under light anesthesia (ketamine (50 mg/kg) and xylazine (5 mg/kg); IP) and fasted overnight, animals kept in head-down positions were given DNBS (0.5 ml; for 30s) through a Teflon cannula (2 mm diameter) inserted 8 cm into the anus. Rats from the non-colitic group intracolonically received 0.5 ml of 0.9% saline. The rats were returned to their cages after the procedure to recover from anesthesia.

Animals from all groups (n= 8) were then euthanized by cervical dislocation under anesthesia (ketamine (80 mg/kg) and xylazine (10 mg/kg); IP) 72 h after colitis induction. Colonic segments were subsequently opened longitudinally after euthanasia. The macroscopic damage score (MDS) was calculated according to the criteria described by Bell et al. (1995). Representative samples were frozen at -80°C and used for determining myeloperoxidase activity (MPO), total glutathione (GSH), malonaldialdehyde content (MDA), cytokine levels, and reverse transcription−quantitative polymerase chain reaction (RT-qPCR). Representative samples were taken from the distal inflamed area for the histological and immunohistochemistry studies.

#### DSS-Induced Colitis: Experimental Design and Treatment Protocol

Male C57BL/6J (n = 32) mice were randomized into four groups (n = 8/groups): control groups (non-colitic and colitic: DSS), and HEBP (100 mg/kg and 200 mg/kg). In this model, mice from the DSS group and the HEBP treated groups received drinking water (*ad libitum*) supplemented with 3% (w/v) DSS for 6 days (Melgar et al., 2005). Mice were treated by oral gavage with the extract or water (control groups) during the experimental period. The animals were then euthanized after 9 days with cervical dislocation under anesthesia (ketamine (80 mg/kg) and xylazine (10 mg/kg); IP). The colonic samples were frozen at -80°C for RT-qPCR analysis. Representative samples were taken from the distal inflamed area for the histological and immunohistochemistry studies.

### Evaluation of DAI and Weight/Length Colonic Relationship on DNBS-Induced and DSS Colitis

The disease activity index (DAI) was determined by combining scores of weight loss, stool consistency and bleeding, as previously described by Cooper et al. (1993). The sum of the points for body weight loss percentage (%) (score 0–4), stool consistency (score 0–4) and rectal bleeding (score 0-4) were calculated from according to Kim et al. (2012). DAI was scored daily after colitis induction in the DNBS and DSS models. The colon was cut near the ileocecal valve in all animals (n = 72), and its length (cm) and weight (g) were measured. The weight/length (g/cm) colonic relationship was then determined.

### Determination of Myeloperoxidase (MPO) Activity, Malonyldialdehyde (MDA) Levels, and Glutathione (GSH) Total in the Intestine

For these analyzes, colonic samples obtained from Wistar rats (model DNBS) were used, cut uniformly and longitudinally, perforated and preserved at -80°C. The MPO activity was measured according to the technique described by Krawisz et al. (1984). The samples (n = 8) were homogenized in 0.5% hexadecyltrimethylammonium bromide (pH = 6.0; 1:20 m/v). The homogenate was centrifuged (2,000×g at 4°C for 20 min) and the supernatant was used to measure MPO activity. Results were expressed as MPO units per gram of wet tissue; one unit of MPO activity was defined as that degrading 1 mmol hydrogen peroxide/min at 25°C. MDA level was measured *via* the assay described by Esterbauer and Cheeseman (1990). Colon samples (n = 8) were suspended in buffer Tris hydrochloride (1:5 w/v) and minced with scissors for 15 s on an ice-cold plate. The resulting suspension was homogenized for 2 min with an automatic Potter homogenizer and centrifuged at 2,500×g at 4°C for 10 min. The supernatants were assayed to determine MDA levels. The results are expressed as nanomoles of MDA per gram of tissue. Total GSH content was measured *via* the assay described by Anderson (1985). The colonic tissue (n = 8) was homogenized with 5% trichloroacetic acid (1:20 w/v). Samples were centrifuged at 10,000×g at 4°C for 15 min. The supernatant was used to measure total GSH content. The results were reported as units of GSH per milligram of tissue. The absorbance of MPO activity, MDA levels and total GSH content were measured at a wavelength (λ) of 450, 586, and 412 nm, respectively.

### Measurement of Cytokine Production in the Intestine

The colonic tissue of Wistar rats was homogenized with phosphate-buffered solution (PBS) (Safieh-Garabedian et al., 1995). IL-1β and TNF-α levels in the rats were determined in colon homogenate supernatants (DNBS model) using commercial enzyme-linked immunosorbent assay (ELISA) kits (R&D Systems, Minneapolis, MN) according to the manufacturer’s instructions. The absorbance was measured at 490 nm and the results were expressed as ng/g of homogenized tissue.

### Analysis of RNA Transcripts by RT-qPCR

Total ribonucleic acid (RNA) in the colon was extracted using a deoxyribonucleic acid (DNA) extraction kit, and its concentration and purity were measured using NanoDrop 2000. Total RNA from colonic samples was reverse transcribed to cDNA and the analysis of gene expression of inflammatory mediators and proteins involved in epithelial integrity was performed through Eco™ PCR real-time optical system (Illumina, San Diego, CA, USA) with specific primers at 10 µM of forward (FW) and reverse (RV) (**Table 1**). mRNA expression was normalized using the housekeeping gene glyceraldehyde 3-phosphate dehydrogenase (GAPDH) and β-actin (Actb) as internal control. The mRNA relative quantification was calculated using the ΔΔCt method.

**Table 1:** Primer sequences used in reverse transcription−quantitative polymerase chain reaction (RT-qPCR) assays for *in vivo* models [2.4-dinitrobenzene sulfonic acid (DNBS) and dextran sulfate sodium (DSS)].

Gene	Organism	Sequence 5’-3’	melting T °C
*Actb*	Rat	FW : CCATCACCATCTTCCAGGAG RV : CCTGCTTCACCACCTTCTTG	60
*GAPDH*	Mouse	FW : CCATCACCATCTTCCAGGAG RV : CCTGCTTCACCACCTTCTTG	60
*IL-1β*	Rat	FW: GATCTTTGAAGAAGAGCCCG RV: AACTATGTCCCGACCATTGC	60
*IL-1β*	Mouse	FW : TGATGAGAATGACCTCTTCT RV : CTTCTTCAAAGATGAAGGAAA	55
*TNF-α*	Rat	FW: GTCTTTGAGATCCATGCCATTG RV: AGACCCTCACACTCAGATCA	57
*TNF-α*	Mouse	FW: AACTAGTGGTGCCAGCCGAT RV: CTTCACAGAGCAATGACTCC	60
*iNOS*	Mouse	FW : GTTGAAGACTGAGACTCTGG RV: ACTAGGCTACTCCGTGGA	67
*IL-6*	Mouse	FW: TAGTCCTTCCTACCCCAATTTCC RV: TTGGTCCTTAGCCACTCCTTCC	60
*OCLUDINA*	Mouse	FW : ACGGACCCTGACCACTATGA RV : TCAGCAGCAGCCATGTACTC	56
*ICAM-1*	Mouse	FW : GAGGAGGTGAATGTATAAGTTATG RV : GGATGTGGAGGAGCAGAG	60
*MUC-2*	Mouse	FW: GCAGTCCTCAGTGGCACCTC RV: CACCGTGGGGCTACTGGAGAG	60
*MUC-3*	Mouse	FW: CGTGGTCAACTGCGAGAATGG RV: CGGCTCTATCTCTACGCTCTC	60
*Z0-1*	Mouse	FW: GGGGCCTACACTGATCAAGA RV: TGGAGATGAGGCTTCTGCTT	56
*TFF-3*	Mouse	FW: CCTGGTTGCTGGGTCCTCTG RV: GCCACGGTTGTTACACTGCTC	60
*VILLIN*	Mouse	FW: TGCTACCTGCTGCTCTATACCTAC RV : CTGGCTCGTCGTTGTACTTCTG	60
*TLR-4*	Mouse	FW: GCCTTTCAGGGAATTAAGCTCC RV: AGATCAACCGATGGACGTGTAA	60
*MCP-1*	Mouse	FW: CAGCTGGGGACAGAATGGGG RV: GAGCTCTCTGGTACTCTTTTG	63

Font: (Vezza et al., 2017).

### Histopathology and Immunohistochemical Analysis

The cross-sections (5 animals per group) of the colonic tissue (inflamed area) were fixed in buffered paraformaldehyde (10% in PBS, pH 7.2) in both experiments, and then processed by histopathological standards and the immunohistochemical technique. Cross-sections were selected and embedded in paraffin. Tissue sections (5 μm) were obtained with a microtome and stained with hematoxylin and eosin for histological evaluation by optical microscopy. The histological microscopic damage index (MIDI) was scored on a scale from 0 to 6, as reported by Zea-Iriarte et al. (1996). Thin colon sections (3 μm) of 5 animals per group were obtained with a microtome and transferred to gelatin-coated slides for immunohistochemical analysis. Each tissue section was then deparaffinised, rehydrated and washed with 0.3% Triton X-100 in PBS, quenched with endogenous peroxidase (3% hydrogen peroxide). The colonic tissue was incubated overnight at 4°C with the following primary antibodies: NF-κB p65 1:100, iNOS 1:500; IL-17 1:800, cyclooxygenase (COX) 2 1:500 (Santa Cruz Biotechnology, Interprise, Brazil). Immunoreactivity was visualized using a colorimetric-based detection kit following the protocol provided by the manufacturer (TrekAvidin-HRP Label + Kit from Biocare Medical, Dako, USA). The cell immunostaining intensity was determined according to Guerra et al. (2015). Histopathological and immunohistochemical analyses were performed independently by two pathologists, blinded to group identity.

### Statistical Analysis

All results were expressed as means ± standard error of the means. Differences between means were tested for statistical significance using one-way ANOVA followed by Tukey test. Non-parametric data (score) are expressed as the median (range) and were analyzed using the Mann-Whitney test. All statistical analyzes were performed using GraphPad 6.1 software (Graph- Pad Software Inc., La Jolla, CA) and statistical significance was set at *p* < 0.05.

## Results

### UFLC-DAD Profile and Quantitative Analyses of Major Flavonoids of the HEBP

**Figure 1:**
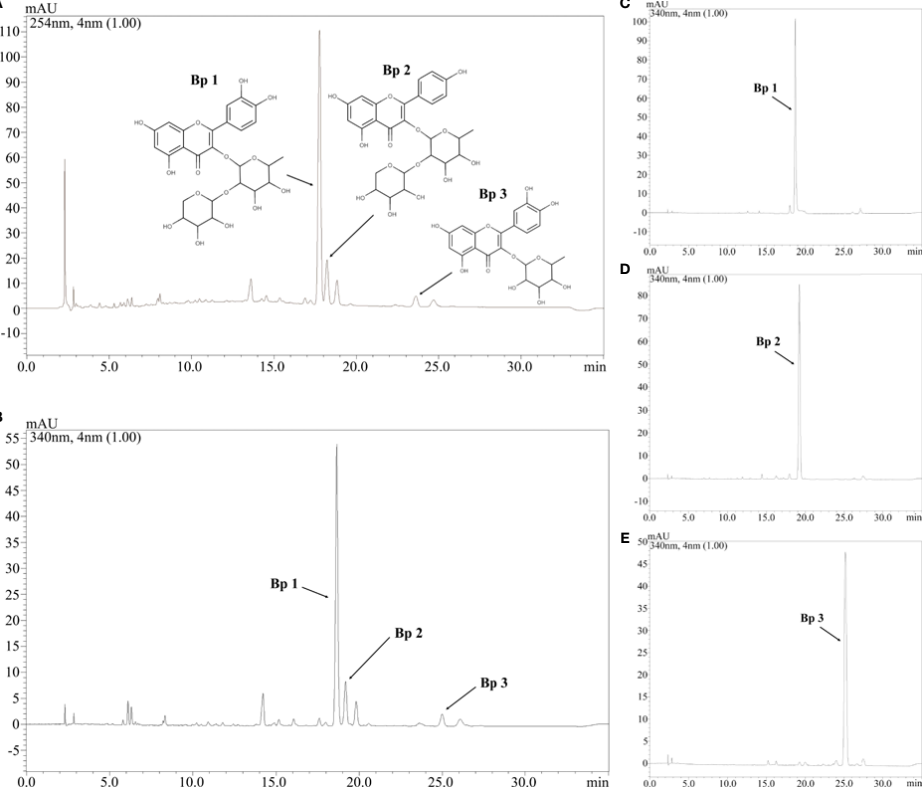
Chromatogram of hydroethanolic extract from *B. pinnatum* leaf (HEBP) at UV 254 **(A)** and 340 **(B)** nm and chromatogram at UV 340 nm of **Bp1 (C)**, **Bp2 (D)**, and **Bp3 (E)**. Stationary phase: C18 Phenomenex (150x4.6 mm, 2.6 μm) equipped with a Phenomenex security guard column (4.0x2.0 mm ID). Mobile phase: trifluoroacetic acid 0.3% and (B) acetonitrile; gradient: 7–15% B, 0–3 min; 15–20% B, 3–12 min; 20–22% B, 12–30 min; flow elution 0.7 ml/min; detection 254 and 340 nm.

**Figure 1** shows the HEBP chromatogram with a main peak and other peaks at UV 254 and 340 nm, corresponding to the flavonoids: **Bp1**, **Bp2**, and **Bp3**. Extract peaks were identified by comparing retention times, UV spectra data and increasing peak areas by co-injection (extract + standard solutions, 1:1, v/v). Most of the peaks found have already been identified and previously described by our research group (Fernandes et al., 2016), but this is the first time that the content of major flavonoids has been quantified and correlated with the pharmacological response. According to our previous study, the major flavonoids identified in the *B. pinnatum* leaf extract by high-performance liquid chromatography coupled with DAD-MS/MS are flavonoids-*O*-glycosides derived from aglicones eupafolin, quercetin, and kaempferol. In this work, the major flavonoid, **Bp1**, and other two compounds **Bp2** and **Bp3** were quantified as possible analytical markers this species. The UFLC-DAD method for the quantification of flavonoids indicated good linearity (r > 0.9997:**Bp1**; r > 0.9996:**Bp2**; r > 0.9988:**Bp3**), specificity, selectivity, precision, robustness and accuracy (**Table 2**). The **Bp1**, **Bp2** and **Bp3** contents were 35.56 ± 0.086 mg, 4.66 ± 0.076 mg and 4.56 ± 0.026 mg, respectively, per g of extract (**Table 2**).

**Table 2:** Validation parameters of the analytical method to quantify the content of **Bp1**, **Bp2**, and **Bp3** *in B. pinnatum* leaf extract at UV 340 nm.

Compounds	System Suitability parameters^a^				Selectivity parameters		Linearity		LOD^c^ (µg/mL)	LOQ^c^ (µg/ml)	Content (mg/g)
	R	T	P	k’	peak purity	*Rt* (min)	r	Calibration curve^b^			
**Bp1**	1.77 ± 0.037	1.42 ± 0.602	51218,33 ± 429.712	7.52 ± 0.278	0.98 ± 0.01	19.20 ± 0.26	0.9997	y = 463320x - 11163	0.0000087	0.0000263	35.56 ± 0.086
**Bp2**	2.11 ± 0.032	1.21 ± 0.035	39904,33 ± 304.733	7.87 ± 0.288	0.95 ± 0.005	19.87 ± 0.28	0.9996	y = 91121x – 789.67	0.0007442	0.0022553	4.66 ± 0.076
**Bp3**	2.82 ± 0.041	1.06 ± 0.005	48178 ± 356.215	10.55 ± 0.378	–	25.58 ± 0.20	0.9997	y = 21351x + 199.57	0.0019840	0.0060120	4.56 ± 0.026

**Compounds**		**Intermediate precision**^d^			**Repeatability**^e^				**Accuracy**^f^		
		**50%**	**100%**	**150%**	**50%**	**100%**	**150%**		**50%**	**100%**	**150%**

**Bp1**	RSD (%)	3.58	3.34	0.56	0.98	0.20	0.48	**Recovery (%)**	97.96	96.24	85.48
**Bp2**		1.74	1.09	1.02	1.11	2.25	0.38		101.18	111.07	77.99
**Bp3**		3.47	3.31	1.45	2.22	2.63	1.71		99.34	110.85	134.99

Legend: R, resolution; T, tail factor; P, column efficiency or theoretical plates; k’, capacity factor; Rt, retention time; r, coefficient of correlation; RSD, relative standard deviation.

a Data obtained through the analysis of peak of compounds **Bp1**, **Bp2**, and **Bp3** from HEBP.

b y = peak area and x = concentration (μg/ml).

c LOD = 3.3R/S and LOQ =10R/S: where R and R are the residual standard deviation of the regression line and slope of the calibration curve, respectively.

d RSD of the mean of the samples analyzed in 3 different days (n = 3).

e RSD of the mean of the samples analyzed on the same day (n = 3).

f Accuracy was determined by the recovery method: were added quantities known of reference substance (**Bp1**, **Bp2** and **Bp3**) to sample (HEBP 2 mg/ml).

### Preventive Effect of HEBP on DNBS-Induced Colitis in Rats

**Figure 2:**
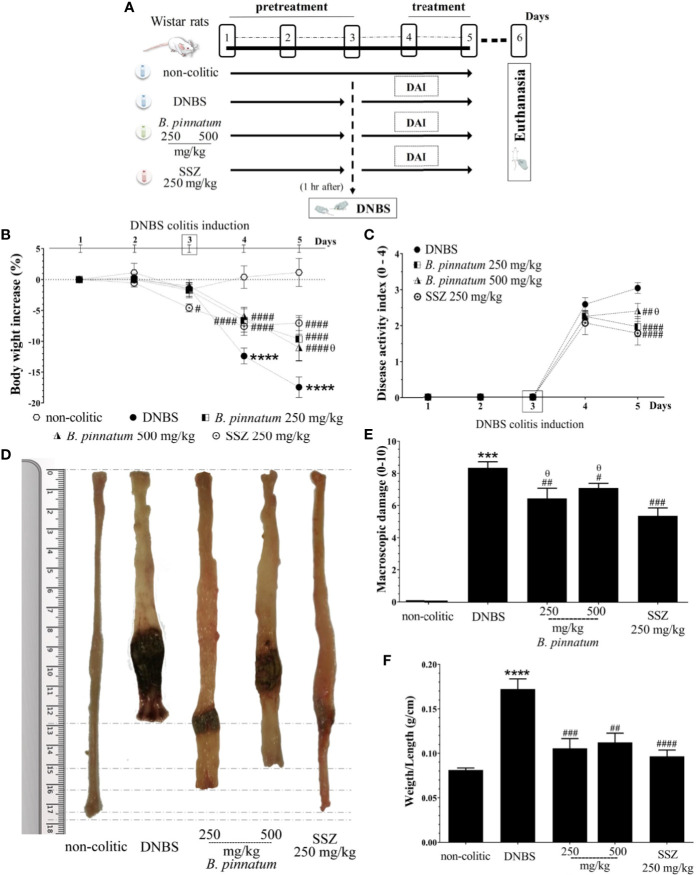
2.4-dinitrobenzene sulfonic acid (DNBS)–induced colitis experimental design **(A)** and effects of treatments with hydroethanolic extract from *B. pinnatum* leaf (HEBP) on weight loss **(B)**, damage of the colon **(C)**, macroscopic damage score (MDS) **(D)**, weight/length ratio **(E)**, and disease activity index (DAI) **(F)**. Data (n = 8) are presented as mean ± standard error from the mean. ***p < 0.001, ****p < 0.0001 compared with non-colitic group; *^#^p* < 0.01, *^###^p* < 0.001, *^####^p* < 0.0001 compared with DNBS group; ^θ^*p* < 0.05 compared with SSZ 250 mg/kg group.

The colonic instillation of DNBS (DNBS-induced colitis on day 3, **Figure 2A**) caused intestinal inflammation, which was evidenced over the course of the experiment by body weight loss (days 4 and 5, **Figure 2B**, *p* < 0.05), the presence of diarrhea and blood in the feces from rats in the DNBS group, parameters evaluated in the DAI (**Figure 2C**). The macroscopic characterization (**Figure 2D**) of the colonic tissue in these rats revealed the presence of an extensive area of inflammation and ulceration, with high MDS values (**Figure 2E**) and a significant increase (*p* < 0.05) in the colonic weight/length ratio (**Figure 2F**) when compared to the non-colitic group. Preventive administration of HEBP (250 and 500 mg/kg) or SSZ (250 mg/kg) had a beneficial effect on intestinal inflammation, evidenced by attenuating body weight loss (day 4) and a reduction in the DAI score (*p* < 0.05), in the MDS (*p* < 0.05) and in the weight/length ratio (*p* < 0.05) of the colon when compared to the DNBS group.

**Figure 3:**
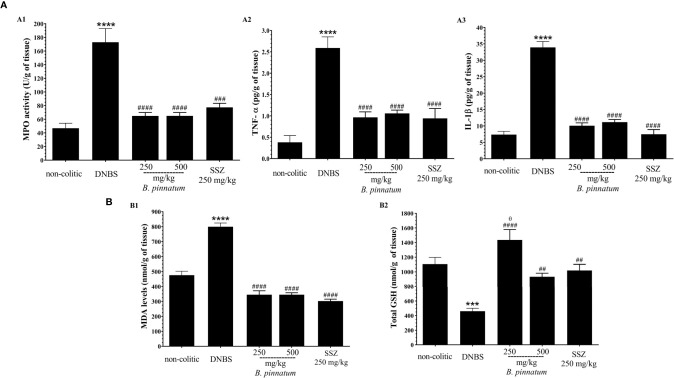
Effect of hydroethanolic extract from *B. pinnatum* leaf (HEBP) on inflammation markers **(A)**: myeloperoxidase activity (MPO) **(A1)**, TNF-α **(A2)**, and IL-1β **(A3)** and oxidative markers **(B)**: malonaldialdehyde content (MDA) **(B1)** and GSH **(B2)** in the intestinal inflammation of the experimental 2.4-dinitrobenzene sulfonic acid (DNBS) model. Data (n = 8) are presented as mean ± standard error from the mean. ***p < 0.001, ****p < 0.0001 compared with non-colitic group; ^#^*p* < 0.05, *^##^p* < 0.01, *^###^p* < 0.001, *^####^p* < 0.0001 compared with DNBS group; ^θ^*p* < 0.05, compared with SSZ 250 mg/kg group.

**Figure 4:**
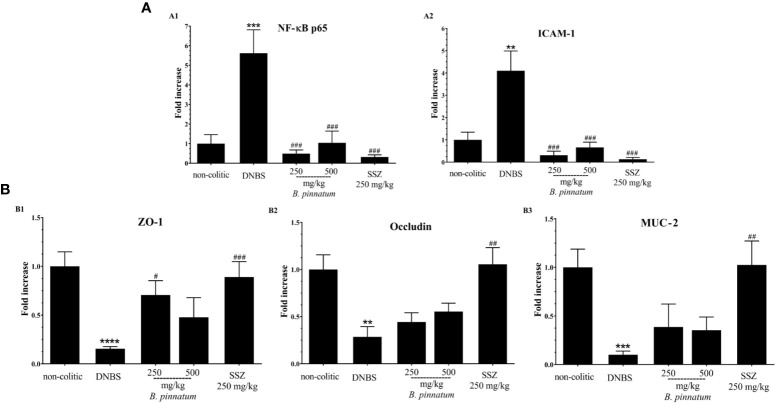
Effect of hydroethanolic extract from *B. pinnatum* leaf (HEBP) on colonic gene expression of different mediators in 2.4-dinitrobenzene sulfonic acid (DNBS)–induced colitis analyzed by reverse transcription−quantitative polymerase chain reaction (RT-qPCR). Inflammation markers **(A)**: NF-κB p65 **(A1)**, ICAM-1 **(A2)**, and markers involved in epithelial barrier integrity **(B)**: ZO-1 **(B1)**, occludin **(B2)**, and MUC-2 **(B3)**. Data (n = 8) are presented as mean ± standard error from the mean. **p < 0.01, ***p < 0.001, ****p < 0.0001 compared with non-colitic group; ^#^*p* < 0.05, *^##^p* < 0.01, *^###^p* < 0.001, compared with DNBS group.

In comparison with the non-colitic group, the colonic inflammation in the DNBS group was associated with increased (*p* < 0.05) MPO activity (**Figure 3A1**), TNF-α (**Figure 3A2**) and IL-1β (**Figure 3A3**) pro-inflammatory cytokine levels, and MDA (**Figure 3B1**), as well as depleted GSH content (**Figure 3B2**). In turn, the anti-inflammatory and antioxidant effect of HEBP (250 mg/kg or 500 mg/kg) and SSZ (250 mg/kg) was also evidenced by a significant improvement (*p* < 0.05) in these markers (MPO, MDA, GSH, IL-1β, and TNF-α) in relation to the DNBS group. Moreover, the colonic damage induced by DNBS (25 mg; intracolonic route) was also characterized by increased (*p* < 0.05 *vs* non-colitic rats) gene expression of NF-κB p65 and ICAM-1 (**Figure 4A1, 2**), as well as by decreased (*p* < 0.05 *vs* non-colitic rats) gene expression of different markers involved in epithelial barrier integrity: ZO-1, occludin and MUC-2 (**Figure 4B1–3**) in the DNBS group. The pre-treatment with HEBP (250 or 500 mg/kg) or SSZ (250 mg/kg) resulted in a significant decrease (*p* < 0.05) in the expression (downregulation) of NF-κB p65 and ICAM-1 in comparison with the DNBS group; as well as in increased expression (upregulation) of ZO-1, occludin and MUC-2 showing significant statistical differences (*p* < 0.05) for ZO-1 with HEBP at doses of 250 mg/kg and with SSZ for all markers. Furthermore, HEBP (250 and 500 mg/kg) did not statistically differ (*p* ≥ 0.05) from SSZ 250 mg/kg in all cases.

**Figure 5:**
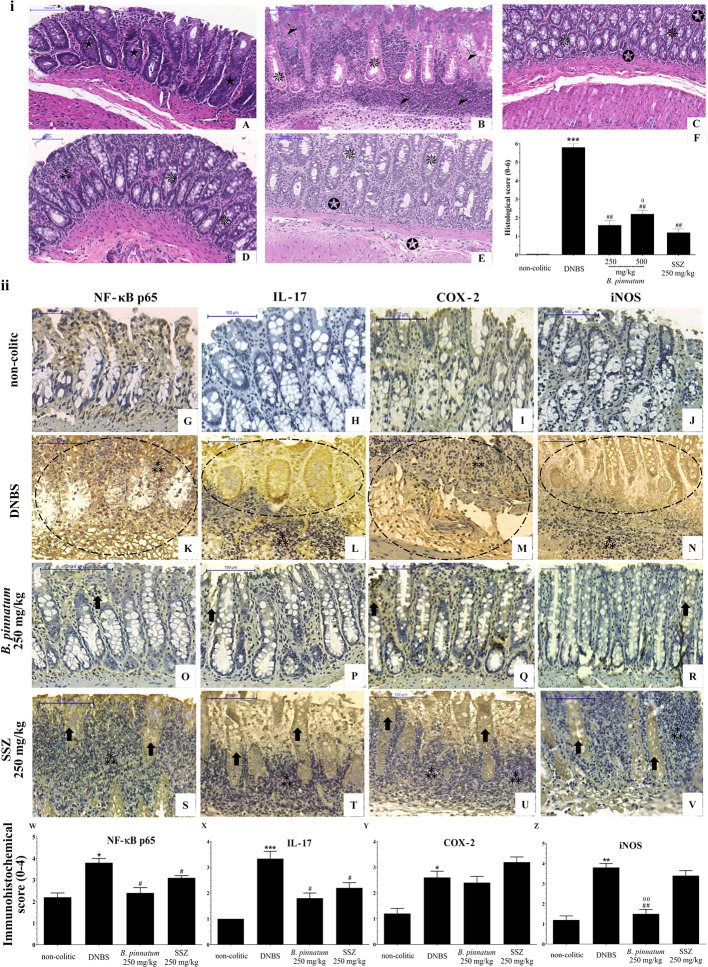
Histopathologic features of representative samples of colonic tissue (**i**), showing the colon fragment cut in the longitudinal direction and stained with Hematoxylin/Eosin; scale 100 μm: non-colitic **(A)**, 2.4-dinitrobenzene sulfonic acid (DNBS) **(B)**, hydroethanolic extract from *B. pinnatum* leaf (HEBP) 250 and 500 mg/kg **(C–D)**, SSZ 250 mg/kg **(E)**, and evaluation of microscopic damage **(F)**. Normal intestinal layers (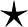), diffused active colitis with superficial erosions, stromal edema, dense acute and chronic inflammatory cells infiltrate with widely (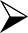), moderate (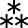), light (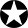) ulcerating mucosa, and loss of goblet cells (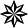). Immunohistochemical analysis of colonic tissue and immunohistochemistry scores (**ii**). Scale bar 100µm. The non-colitic **(G-J)**, DNBS **(K–N)**, HEBP 250 mg/kg **(O–R)** and SSZ 250 mg/kg **(S–V)** groups were analyzed by immunoreactivity for NF-κB p65, IL-17, COX-2, and iNOS. Arrows indicate antibody reactivity, 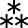 indicates area of intense inflammatory cell infiltration, dashed areas indicate ulceration. Data (n = 8) are presented as mean ± standard error from the mean. *p < 0.05, **p < 0.01, ***p < 0.001, compared with non-colitic group; ^#^*p* < 0.05, *^##^p* < 0.01, compared with DNBS group; ^θ^*p* < 0.05, ^θθ^*p* < 0.01, compared with SSZ 250 mg/kg group.

The histological evaluation (**Figure 5i**) of the colonic specimens from DNBS assay confirms the anti-inflammatory activity of HEBP and SSZ. Intense transmural inflammation was observed when comparing the colon structure of the non-colitic group (**Figure 5A**) with the DNBS group (**Figure 5B**), comprising the mucosa and submucosa layers with extensive superficial ulceration, resulting in almost complete destruction of the crypts, absence of goblet cells and presence of edema. Treatment with HEBP (250 and 500 mg/kg; **Figures 5C, D**) and SSZ (250 mg/kg; **Figure 5E**) attenuated this DNBS-induced colonic damage, reduced inflammatory infiltrate and edema, and promoted epithelial regeneration, as evidenced by reduced MIDI (**Figure 5F**, *p* < 0.05). The immunohistochemistry data (**Figure 5ii**) corroborate the findings described above, showing that DNBS caused an increase in immunoreactivity for NF-κB p65, IL-17, COX 2, and iNOS mediators (**Figures 5G–V**). On the other hand, the groups subjected to treatment with HEBP 250 mg/kg and SSZ 250 mg/kg showed a decrease in immunostaining of NF-κB p65, IL-17, COX-2, and iNOS, which was confirmed by the immunohistochemical score (**Figures 5W-Z**, *p* < 0.05).

### Effect of HEBP on DSS-Induced Colitis in Mice

**Figure 6:**
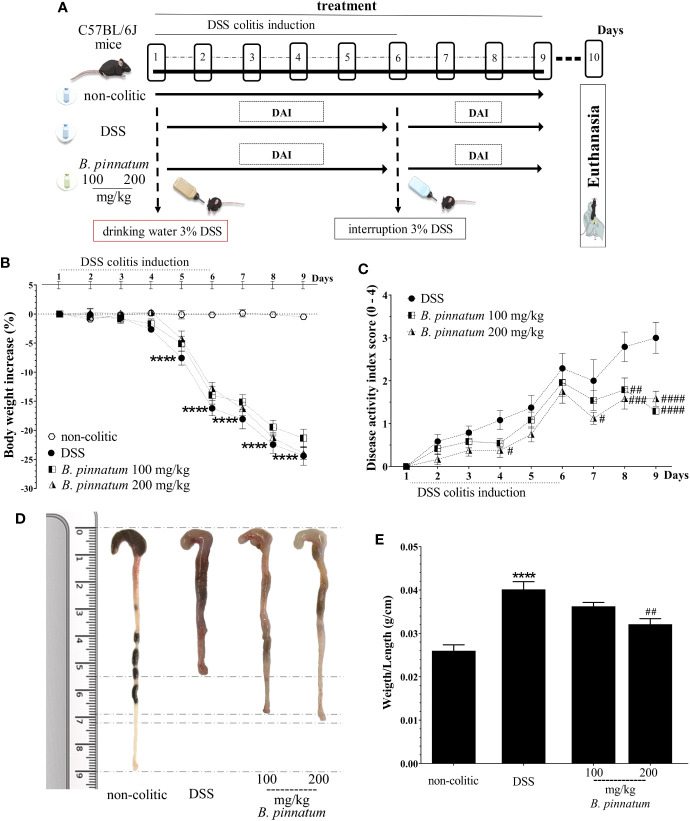
Dextran sulfate sodium (DSS)–induced colitis experimental design **(A)** and effects of treatments with hydroethanolic extract from *B. pinnatum* leaf (HEBP) on weight loss **(B)**, disease activity index (DAI) **(C)**, damage **(D)**, and weight/length ratio **(E)** of the colon. Data (n = 8) are presented as mean ± standard error from the mean. ****p < 0.0001 compared with non-colitic group; ^#^*p* < 0.05, *^##^p* < 0.01, *^###^p* < 0.001, *^####^p* < 0.0001 compared with DSS group.

After colitis induction (day 1, **Figure 6A**), the daily weight, food intake, and water consumption of mice from each group were recorded and analyzed in the DSS experiment. The non-colitic group had normal food and drinking water intake, and their body weight showed an irrelevant variation. DSS (3% in drinking water; w/v) intake caused weight loss (**Figure 6B**, *p* < 0.05 *vs* non-colitic group) in the mice of the DSS and HEBP (100 and 200 mg/kg) groups from day 4, which was associated with diarrhea and the presence of blood in the feces. This resulted in increased DAI values (**Figure 6C**) over time. However, DSS-colitic mice treated with HEBP at doses of 100 or 200 mg/kg significantly reduced (*p* < 0.05) the DAI score in the days following the interruption of DSS administration (day 6) when compared to the DSS group. The colon was isolated and its length was measured with a ruler and inflammation and ulceration areas was analyzed (**Figure 6D**). The results showed that the colon was longer in the mice from the non-colitic group than in the mice from the DSS group. The extract (100 and 200 mg/kg) attenuated the colon shortening and decreased the inflammation and ulceration areas in the DSS-induced group. No significant differences (*p* ≥ 0.05) were observed in the weight/length ratio (**Figure 6E**) of the colon when compared to the DSS group once the mice were sacrificed, although a decreasing trend was observed with the higher assayed dose.

**Figure 7:**
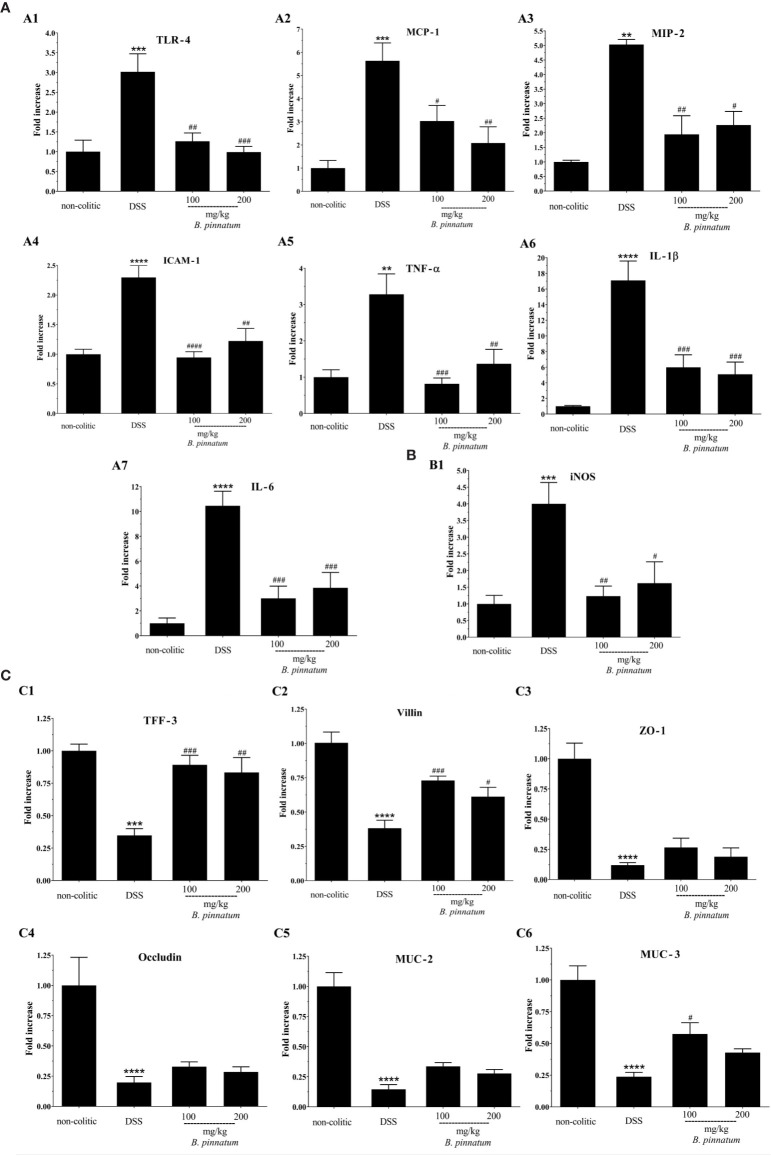
Effect of hydroethanolic extract from *B. pinnatum* leaf (HEBP) on colonic gene expression of different mediators in dextran sulfate sodium (DSS)–induced colitis analyzed by reverse transcription−quantitative polymerase chain reaction (RT-qPCR). Inflammation markers **(A)**: TLR-4 (**A1**), MCP-1 (**A2**), MIP-2 (**A3**), ICAM-1 (**A4**), TNF-α (**A5**), IL-1β (**A6**), IL-6 (**A7**), oxidative marker **(B)**: iNOS (**B1**) and markers involved in epithelial barrier integrity **(C)**: TFF-3 (**C1**), villin (**C2**), ZO-1 (**C3**), occludin (**C4**), MUC-2 (**C5**), and MUC-3 (**C6**). Data (n = 8) are presented as mean ± standard error from the mean. **p < 0.01, ***p < 0.001, ****p < 0.0001 compared with non-colitic group; ^#^*p* < 0.05, *^##^p* < 0.01, *^###^p* < 0.001, *^####^p* < 0.0001 compared with DSS group.

In comparison with non-colitic mice, DSS colitis was associated with changes in the intestinal immune response, as evidenced by the increased (*p* < 0.05) colonic expression of different pro-inflammatory markers, including Toll-like receptor 4 (TLR-4), chemokines (MCP-1 and MIP-2), ICAM-1, cytokines (TNF-α, IL-1β, IL-6), and iNOS (**Figures 7A1–7, B1**). Treatment with HEBP (100 and 200 mg/kg) in colitic mice reduced (*p* < 0.05) the gene expression of all these inflammatory and oxidative markers (TLR-4, MCP-1, MIP-2, ICAM-1, TNF-α, IL-1β, IL-6, and iNOS) in comparison to the DSS group. In addition, DSS-induced colitis compromised the intestinal barrier function, as shown by the decreased (*p* < 0.05) expression of different proteins involved in maintaining epithelial integrity, including TFF-3, villin, ZO-1, occludin, MUC-2, and MUC-3 (**Figures 7C1–6**) when compared to the non-colitic group. The administration of HEBP to colitic mice increased (p < 0.05 *vs* DNBS group) the colonic expression of TFF-3 and villin when compared to the DSS group. An increasing trend was observed in the expression of ZO-1, occluding, and MUC-2 (*p* ≥ 0.05 *vs* DNBS group) and MUC-3 expression was significantly increased (p < 0.05 *vs* DNBS group) by HEBP administration at the 100 mg/kg dose.

**Figure 8:**
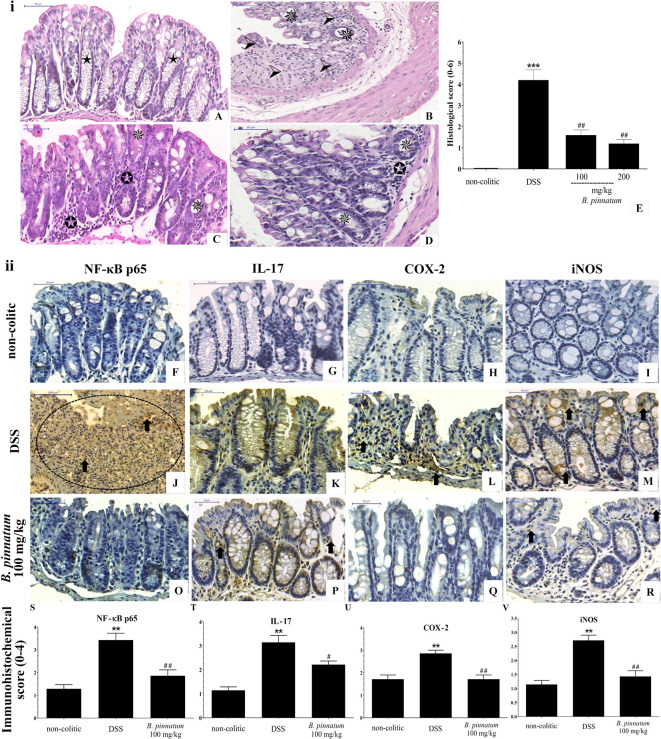
Histopathologic features of representative samples of colonic tissue (**i**), showing the colon fragment cut in the longitudinal direction and stained with Hematoxylin/Eosin; scale 50 μm: non-colitic **(A)**, DSS **(B)**, HEBP 100 and 200 mg/kg **(C, D)**, and the evaluation of microscopic damage **(E)**. Normal intestinal layers (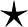), diffused active colitis with superficial erosions, stromal edema, dense acute and chronic inflammatory cells infiltrate with widely (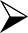), moderate (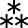), light (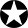) ulcerating mucosa, and loss of goblet cells (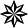). Immunohistochemical analysis of colonic tissue and immunohistochemistry scores (**ii**). Scale bar 50µm. The non-colitic **(F**–**I)**, DSS **(J–M)**, and HEBP 100 mg/kg **(O**–**R)** groups were analyzed by immunoreactivity for NF-κB p65, IL-17, COX-2, and iNOS. Arrows indicate antibody reactivity, 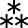 indicates area of intense inflammatory cell infiltration, dashed areas indicate ulceration. Data (n = 8) are presented as mean ± standard error from the mean. **p < 0.01, ***p < 0.001, compared with non-colitic group; ^#^*p* < 0.05, *^##^p* < 0.01, compared with DSS group.

The histological (**Figure 8i**) analysis and immunostaining (**Figure 8ii**) of the NF-κB p65, IL-17, COX-2, and iNOS antibodies reinforced the pre-clinical efficacy of the HEBP extract in the DSS model. When comparing to the colon structure of the non-colitic group (**Figure 8A**) the colonic specimens from the DSS group (**Figure 8B**) showed intense transmural inflammation, which affected the mucosa and the submucosa. Lesions were found in the colonic tissue, including extensive mucosal ulceration and almost complete destruction of the crypts, absence of goblet cells, and edema formation were observed in these mice from the DSS group. Administrating extract (HEBP 100 and 200 mg/kg, **Figures 8C, D**) to colitic mice resulted in improving the histological damage induced by DSS, as characterized by epithelial regeneration, decreased inflammatory infiltrate and edema. These data were corroborated by histopathological score (**Figure 8E**, *p* < 0.05 *vs* DSS group). In addition, the HEBP (100 and 200 mg/kg) decreased immunoreactivity of NF-κB p65, IL-17, COX-2, and iNOS proteins (**Figures 8F–R**) as well as the immunohistochemical scores (**Figures 8S-V**, *p* < 0.05 *vs* DSS group).

## Discussion

Human IBD (CD and UC) are characterized by abnormal activation of the gut immune system, which results in local chronic inflammation (Barone et al., 2018). The experimental models (DNBS and DSS) used in this study predict results in humans and are crucial to consider the real-life context of the disease. The present research reveals that HEBP exerts intestinal anti-inflammatory effects on experimental colitis models in rodents, since it was able to prevent the colonic damage induced by DNBS in rats and to improve the DSS-associated colonic inflammation in mice. These effects were evidenced by improvement in the different parameters evaluated in the DAI (diarrhea, weight loss and the presence of blood in feces), as well as of the intestinal macroscopic and histopathological damage. The main compounds previously identified in the HEBP (a potential phytotherapeutic plant extract) were quercetin- and kaempferol-derived flavonoid glycosides (Muzitano et al., 2006; Cruz et al., 2012; Fernandes et al., 2016). These flavonoids showed beneficial effects on intestinal inflammation in the same experimental colitis models (DNBS and DSS) (Salaritabar et al., 2017).

In fact, both DNBS (intracolonic route) and DSS (orally) models are considered adequate to study the immunoregulatory pathways in intestinal inflammation, which are closely linked to the pathogenesis of IBD (Valatas et al., 2013). In this sense, one of the main mechanisms involved in the intestinal inflammatory process is T cell activation (Valatas et al., 2013). Regulation of the differentiation and proliferation of inflammatory Th in the Th1, Th2 and Th17 subgroups can be initiated by TLR activation (especially TLR-4) after rupture of intestinal homeostasis (Lu et al., 2018). TLR are transmembrane proteins (glycoproteins) and are expressed in cells of both the innate and adaptive immune systems (monocytes, macrophages, lymphocytes, mast cells, dendritic cells), as well as in the intestinal epithelium (Sipos, 2014).

Activation of TLR by components which induce the immune response (i.e. DNBS and DSS) causes recruitment of interleukin-1 receptor-associated kinase 1 (IRAK-1) and 4 (IRAK-4) which stimulate tumor necrosis factor receptor-associated factor 6 (TRAF6). This excites the growth factor beta-activated kinase 1 (TAK1), which in turn generates activation of the IKK kinase complex. Such a complex promotes phosphorylation and degradation of IκB-α (Inhibitor *kappa* B-alpha), resulting in the dissociation and release of NF-κB p65, known as an important factor in regulating this highly expressed immune process in patients with IBD (Trzeciak-Jędrzejczyk et al., 2017; Yao et al., 2017; Lu et al., 2018). The NF-κB p65 migrate to the nucleus of cells and downregulate or supra-regulate the expression genes associated with the transcription of different inflammatory mediators (IL-12, IL-18, IL-23, IL-6, IL-1β, IL-10, TNF-α, and prostaglandins), activator protein 1, granulocyte-macrophage colony-stimulating factor, intercellular adhesion molecules, and inflammatory enzymes (iNOS and COX) (Yao et al., 2017; Ramos and Papadakis, 2019).

Based on this information, treatment with HEBP (orally) was able to prevent the abnormal increase of colonic TLR-4 expression, probably by limiting the activation of the subsequent signaling cascade, i.e., it decreased mRNA expression and immunoreactivity of NF-κB p65, IL-17 and COX-2. In turn, this downregulation may be associated with a reduction in some of the evaluated pro-inflammatory cytokines (TNF-α, IL-1β and IL-6). The inhibition of the inflammatory response exerted by HEBP in DNBS- and DSS-induced colitis is most probably due to the presence of the phenolic compounds in the extract, which directly or indirectly prevent the activated inflammatory pathways: TLR-4, NF-κB p65, COX-2, or IL-17, thus contributing to restoring the intestinal homeostasis. In fact, inhibition in the production of TLR-4, NF-κB p65, COX-2, IL-17, TNF-α, IL-1β, and IL-6 is considered useful in treating colitis (Bribi et al., 2016; Ramos and Papadakis, 2019). It is worth highlighting the role of IL-6, which is a pro-tumorigenic cytokine which affects cell proliferation, survival, differentiation, and migration; in fact, the inhibition of IL-6 not only reduces intestinal inflammation, but is also associated with decreased risk of colorectal cancer in IBD patients (Waldner and Neurath, 2014).

In experimental colitis models, as well as in human IBD, the intestinal inflammatory process is directly associated with the induction of oxidative stress, which reduces the cellular antioxidant capacity. The excessive production of free radicals can react with the fatty acids of the cell membrane, in turn leading to lipid peroxidation, and thus contributing to intestinal oxidative damage (Safdari et al., 2016; Nikkhah-Bodaghi et al., 2019). As a result, another mechanism which could justify the beneficial effects of HEBP on these experimental colitis models can be associated with the antioxidant properties which it may exert, mostly due to the presence of flavonoids, especially quercetin and kaempferol. This effect was evidenced by the increase in the colonic content of the antioxidant peptide GSH and reduced MDA levels, a lipid peroxidation marker.

Moreover, it has been reported that excessive production of NO in IBD causes deleterious effect to the tissue through the production of ROS and RNS which are responsible for cytotoxic processes, such as lipid peroxidation and DNA damage, thus resulting in inflammation and tissue damage (Andrade et al., 2018; Simioni et al., 2018). The results of the present study revealed that HEBP reduced the gene expression and immunoreactivity of iNOS in the colonic tissue of treated colitic mice. All these antioxidant effects can play an important role in improving the pathogenic process which involves neutrophil infiltration, and in turn ROS and RNS overproduction in patients with IBD (Salaritabar et al., 2017).

The intensity and maintenance of the inflammatory response, both in clinical and experimental IBD, are also determined by coordinated mechanisms of cell recruitment, which comprise the positive regulation of the expression of intercellular adhesion molecules (ICAM-1) and chemokines (MIP-2 and MCP-1), mainly by activated NF-κB p65 (Trzeciak-Jędrzejczyk et al., 2017; Wang et al., 2018). Colonic inflammation in this study was associated with increased mRNA expression of MIP-2, MCP-1 and ICAM-1, since these proteins recruit defense cells (leukocytes) and favor their adhesion to the activated endothelium at the inflammation site (Vainer, 2005; Abiodun et al., 2016). The downregulation in the expression of the MIP-2 and MCP-1 chemokines, and of the ICAM-1 adhesion molecule by the HEBP improved colonic inflammation in both experimental colitis models, thus resulting in reduced migration and penetration of inflammatory cells into the intestinal mucosa. This would explain the reduction in inflammatory infiltrate and its deleterious impact on the intestinal tissue observed in histological examinations.

Immune deregulation and the generation of ROS in IBD may also be associated with the functional impairment of IEC (enterocytes, goblet cells, neuroendocrine cells, Paneth cells, and M cells). The expression and production of inflammatory mediators alters the integrity and functionality of these cells, promoting a rupture in the barrier of tight epithelial junctions and imbalance in paracellular permeability (Vezza et al., 2017; Ramos and Papadakis, 2019). This was evidenced by decreased TFF-3, ZO-1, and occludin expression, and aggravated by the reduction of MUC-2 (the main constituent of the mucus layer in the colon), MUC-3 (a membrane-bound mucin) and villin-1 in the colitic control group of the tested experimental models. The gene expression of the proteins associated with mucosa protection was slightly upregulated by the HEBP treatment, presenting an increase which is relevant in TFF-3, MUC-3, and villin-1 proteins. In fact, TFF-3 and villin-1 are considered as parameters for goblet cell function, which play an important role in the protection, restitution and healing of the mucosa (Vezza et al., 2017), and highlight another potential pharmacological property of the HEBP.

The treatment of colitis animals using different HEBP doses resulted in improving most of the pro-inflammatory cytokines and intestinal barrier proteins evaluated, revealing the contribution of the immunomodulatory properties of the different flavonoids present in the extract. Pretreatment with the extract in the DNBS model enabled a protective condition in the animal’s body against the development of the disease. Several investigations have reported that one of the initial stages of IBD is related to interruption of the barrier in the intestinal mucosa which results in inflammation and dysregulation of mucosal homeostasis. When this occurs, cells involved in innate immunity like macrophages and dendritic cells can activate PRRs in epithelial cells, including TLR (Mogensen, 2009). It can be proposed that one of the action mechanisms of HEBP in IBD involves its ability to decrease the gene expression of TLR-4, which reduces the deregulated production of pro-inflammatory cytokines (COX-2, IL-17, TNF- α, IL-1β, and IL-6), adhesion molecules (ICAM-1), and chemokine (MIP-2 and MCP-1) due to the down-regulation of NF-κB p65.

These cytokines promote disruption of epithelial integrity, facilitating access of the antigen to the submucosa, the activation of immune cells, and the infiltration of leukocytes in the inflamed tissue (Bribi et al., 2016; Yousefi et al., 2019). Infiltration of activated leukocytes favors production and release of more pro-inflammatory cytokines, thus playing a fundamental role in inducing oxidative reactions in the intestinal mucosa. Another action mechanism which can be proposed is that the extract has the capacity to reduce leukocyte infiltration, and together with its antioxidant property manages to maintain or recover the integrity of the already compromised intestinal barrier. Thus, HEBP has a fundamental role in regulating intestinal immune homeostasis and the inflammatory responses. The results obtained in the DNBS and DSS models indicate that HEBP has both chemopreventive and anti-inflammatory effects.

Preclinical studies with intestinal inflammation models conducted with plant extracts (e.g. *Passiflora subpeltata*, *Citrus aurantium*, *Ipomoea asarifolia*) which contain phenolic compounds derivatives (gallic acid, apigenin, catechin, luteolin, quercetin, alpinetin, rutin) showed that their extracts have significant intestinal anti-inflammatory effects due to their ability to suppress levels of inflammatory and oxidative mediators (da Silva et al., 2018; He et al., 2019; Shanmugam et al., 2020). Park et al. (2012); Sotnikova et al. (2013); Mascaraque et al. (2015); Carrera-Quintanar et al. (2018); Duan et al. (2020) and Vezza et al. (2016) report that flavonoids (among them quercetin and kaempferol) have shown remarkable effects in the attenuation of intestinal inflammation in pharmacological studies (e.g. DNBS- and DSS-model) in animals (rodents) and in clinical studies. As in the present study, the researchers reported that the animals treated (pre and/or post treatment) with a flavonoid- rich extract had significantly lower DAI scores, a reduction in multiple tissue injuries and ulceration, in the infiltration of inflammatory cells and in the shortening of the crypts.

According to researchers, flavonoids suppressed the activation of TLR-4/NF-κB p65 signaling pathway, leading to a decrease in gene expression of TNF-α, IL-1B, and IL-6, COX-2, TFF-3, and iNOS proteins in the intestinal mucosa in colitis model (Park et al., 2012; Sotnikova et al., 2013; Vezza et al., 2016; Duan et al., 2020). Previous reports also demonstrated the potential effects of treatment with *B. pinnatum* leaf extracts as antioxidant and immunoregulator, promoting down-regulation inflammatory markers (IL-6, IL-1β, TNF-α, iNOS, NF-κB p65, and COX-2) in animal models for allergic airway disease (Cruz et al., 2012), acute, and chronic skin inflammation (Chibli et al., 2014), local inflammation induced by *Bothrops jararaca* snake venom (Fernandes et al., 2016) and showing a protective effect on gastric lesion (Sobreira et al., 2017; de Araújo et al., 2018). The authors associated the pharmacological response to the flavonoids present in the extracts.

In summary, this study provides pre-clinical evidence of the intestinal anti-inflammatory effect of HEBP when administered preventively (DNBS model) and in post-treatment curative protocols (DSS model). HEBP modulated the inflammatory response, reduced the expression of important pro-inflammatory markers, decreased oxidative stress, and improved the integrity of the intestinal barrier while also maintaining the cytoarchitecture of colonic tissue. The beneficial effect of HEBP can be attributed to the presence of quercetin- and kaempferol-derived flavonoids glycosides, making it a potential therapeutic alternative in the control of human IBD.

There are currently several therapeutic strategies available to treat IBD. However, most treatments can be associated with serious adverse effects. This justifies the search for new treatments which can combine effectiveness with a low risk of adverse effects (Samsami-kor et al., 2015). In this sense, we highlight the interest in herbal products rich in bioactive metabolites, especially phenolic compounds which are recognized by their immunomodulatory and antioxidant properties, as is the case of HEBP. This extract showed relevant pharmacological effects in preclinical assays. In view of this, there is the potential to conduct some future clinical and about action mechanism of compounds **Bp1**, **Bp2** and **Bp3** studies to ensure safe and effective development of a phytotherapeutic treatment.

## Data Availability Statement

The raw data supporting the conclusions of this article will be made available by the authors, without undue reservation.

## Ethics Statement

The animal study was reviewed and approved by Universisty of Granda (No. CEEA-2010-286) and Federal University of Rio Grande do Norte (CEUA N°26/2016 and N°60/2017).

## Author Contributions

AWLA, DA, JF, TC, and PD-E performed the investigation and analyzed the data. GG, JG, MR-C, and SZ conceived and designed the experimental tests. LH-G helped in the execution of research. GG, RA, AAA, MR-C, JG, and SZ contributed reagents/materials/analysis. The paper was written and reviewed by AWLA, GG, MR-C, JG, and SZ.

## Funding

This study was financed by the *Coordenação de Aperfeiçoamento de Pessoal de Nível Superior - Brasil (CAPES)* and by the *Junta de Andalucía* (CTS 164) and by the Spanish Ministry of Economy and Competitiveness (AGL2015-67995-C3-3-R) with funds from the European Union. AWLA is a predoctoral fellow from Federal University of Rio Grande do Norte - UFRN (“*Programa de Pós-Graduação em Desenvolvimento e Inovação Tecnológica em Medicamentos*”). PD-E and LH-G are predoctoral fellows from University of Granada (“*Programa de Doctorado: Medicina Clínica y Salud Pública*” B12.56.1). The CIBER-EHD is funded by the *Instituto de Salud Carlos III*.

## Conflict of Interest

The authors declare that the research was conducted in the absence of any commercial or financial relationships that could be construed as a potential conflict of interest.
